# The Synergism of β‐Cyclodextrin and Fe^3+^ Enabled Anti‐Swelling Ion‐Conductive Hydrogels for Multimodal Underwater Sensing Aided by Machine Learning Algorithms

**DOI:** 10.1002/advs.74916

**Published:** 2026-03-28

**Authors:** Hao Dong, Chuanliu Liu, Yiming Luo, Dongyu Si, Menghan Zhao, Fangyu Zhao, Yixue Zhang, Wei Zeng, Eman Ramadan Elsharkawy, Terence Xiaoteng Liu, Zeinhom M. El‐Bahy, Hua Hou, Zhanhu Guo, Huige Wei

**Affiliations:** ^1^ State Key Laboratory of Bio‐Based Fiber Materials Tianjin Key Laboratory of Brine Chemical Engineering and Resource Eco‐Utilization College of Chemical Engineering and Materials Science Tianjin University of Science and Technology Tianjin China; ^2^ Center for Health Research Northern Border University Arar Saudi Arabia; ^3^ Faculty of Engineering and Environment Northumbria University Newcastle Upon Tyne UK; ^4^ SKKU Global Research Center (SGRC) Sungkyunkwan University (SKKU) Suwon 16419 Republic of Korea; ^5^ College of Materials Science and Engineering Taiyuan University of Science and Technology Taiyuan China; ^6^ Department of Mechanical and Construction Engineering Faculty of Engineering and Environment Northumbria University Newcastle upon Tyne UK

**Keywords:** anti‐swelling, information transfer, machine learning algorithms, underwater sensing

## Abstract

As the key sensing component of flexible sensors, conductive hydrogels are prone to mechanical property degradation and signal deterioration owing to water absorption and swelling in underwater environments. Herein, an anti‐swelling ion‐conducting hydrogels are designed by incorporating ferric chloride (FeCl_3_) and β‐cyclodextrin (β‐CD) into acrylamide‐acrylic acid copolymer (MA) via a one‐pot method, short for MAF*_x_* D*_y_*. Thanks to the synergism of β‐CD and Fe^3+^, the swelling ratio of MAF*_x_* D*_y_* is significantly reduced comparted to its counterparts, for example, 4% for MAF_0.2_D_0.3_, compared to 760% for MAF_0.2_ hydrogel without β‐CD and 526% for MAD_0.3_ without Fe^3+^ after soaking in deionized water for 30 days. Notably, the multimodal underwater sensor constructed based on the MAF_0.2_D_0.3_ hydrogel can not only monitor underwater human joint motion but also realizes the accurate transmission of underwater information with the help of Morse code. Moreover, aided by machine learning algorithms, this study proposes a novel shortcut key recognition strategy to achieve precise and rapid information transmission via letter recognition (with a recognition accuracy of 98.6%), dramatically shortening the time required for underwater communication. This study provides innovative solutions by the rational design of anti‐swelling ion‐conducting hydrogels for reliable and fast underwater communications.

## Graphical abstract

An anti‐swelling ion‐conductive hydrogel is developed via β‐cyclodextrin and Fe^3^^+^ synergism, enabling reliable underwater motion sensing and Morse code communication. Integrated with machine learning, the system achieves 98.6% accuracy in real‐time underwater handwriting recognition for rapid information transmission.

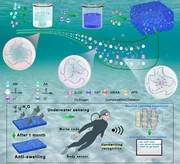

## 1 Introduction

With the rapid development of artificial intelligence, the Internet of Things and wearable electronics technology, flexible sensors have been rapidly developed in recent years in the fields of artificial intelligence, wearable electronics, health monitoring, gesture recognition, and human–computer interaction [1, 2]. Compared with traditional rigid sensors, flexible sensors exhibit better adaptability, enabling perfect integration with complex or curved surfaces and human skin while remaining highly wearable and comfortable [3, 4, 5]. In particular, in the application scenarios of biomedical monitoring [6], sports health management [7], smart home [8], and Internet of Things [9], the advantages of flexible sensors are becoming increasingly significant, and flexible sensors have become an important part of many high‐tech products and solutions [10, 11, 12].

Conductive hydrogels are promising candidates used for the electrode materials of flexible sensors. Current research on conductive hydrogels focuses on enhancing the sensing performance through multiphase composite systems, mainly including three types of functional materials: conductive polymer systems (PPy [13], PEDOT:PSS [14], and PANI [15]), carbon‐based materials (MXene [16], graphene [17], and carbon nanotubes [18]), and metal nanomaterials [19]. Embedding these conductive fillers into hydrogel networks remarkably improves the sensitivity and shortens the response times of sensors [20]. With the expansion of human underwater activities, conductive hydrogels face unprecedented opportunities as well as challenges (e.g., signal drift, mechanical degradation) [21, 22]. On one hand, their excellent biocompatibility and conductive properties provide an ideal platform for underwater human–computer interaction. On the other hand, complex underwater environments severely challenge their reliability [23, 24]. A core challenge stems from the inherent swelling properties of hydrogels. The strong hydrogen bonding between the hydrophilic polymer network and water molecules leads to an osmotic pressure imbalance, whereas the elastic modulus of the traditional crosslinked network is difficult to effectively counteract this pressure difference and ultimately triggers the decay of the mechanical properties and the drift of the sensing signals, restricting their underwater applications [25]. To address this key scientific problem, various innovative solutions have been proposed. Wu et al. [26] used a structural strengthening strategy to construct an SBMA/TA/PVA dual‐network hydrogel, which significantly improved the anti‐swelling performance through the synergistic effect of physical and chemical cross‐linking. Lei et al. [27] developed a hydrogel with multiple cross‐linking structure through the triple mechanism of hydrogen bonding, electrostatic interactions and metal–ligand bonding, which maintained a stable morphology in multiple solvent environments and was successfully applied to underwater motion detection and communication systems. Guan et al. [28] used solvent replacement technology to prepare an environmentally resistant hydrogel, which increased the mechanical stress by 119.62% after being immersed in 0.6 m NaCl for 15 d. Unfortunately, the preparation of the above‐mentioned solubilization‐resistant hydrogels typically involves lengthy and complex methodologies. The development of innovative hydrogels with both rapid prototyping capability and environmental stability still remains challenging.

In this study, a rapid preparation of MAF*_x_* D*_y_* hydrogels was achieved by integrating ferric chloride and β‐cyclodextrin (β‐CD) into an acrylamide‐acrylic acid copolymer (MA) via a one‐pot method. The introduction of Fe^3+^ accelerates the cross‐linking process of the hydrogel through the formation of complexation with the anionic groups of β‐CD and MA. Meanwhile, β‐CD contains multiple hydroxyl groups that can interact with the polymer chain segments through multiple hydrogen bonds, Van der Waals forces, and other intermolecular forces, effectively enhancing the network crosslinking density and conferring excellent anti‐swelling properties to the hydrogel. Notably, the multimodal underwater sensor constructed based on the MAF_0.2_D_0.3_ hydrogel can not only monitor underwater human joint motion but also realizes the accurate transmission of underwater information with the help of Morse code. Simultaneously, this study proposes a novel shortcut key recognition strategy that is combined with machine learning algorithms, to achieve high‐precision analysis of underwater handwriting trajectories and to realize rapid information transmission via letter recognition, which dramatically shortens the time required for underwater communication. This study effectively solves the problem of underwater degradation of traditional hydrogel sensors and provides an important material design paradigm for the development of a new generation of underwater flexible electronic devices, which has promising prospects in the fields of marine exploration equipment, underwater wearable human–machine interface, and underwater intelligent emergency rescue.

## 2 Results and Discussion

### 2.1 Design Strategy and Synthesis of MAF*_x_* D*_y_* Hydrogels

**FIGURE 1:**
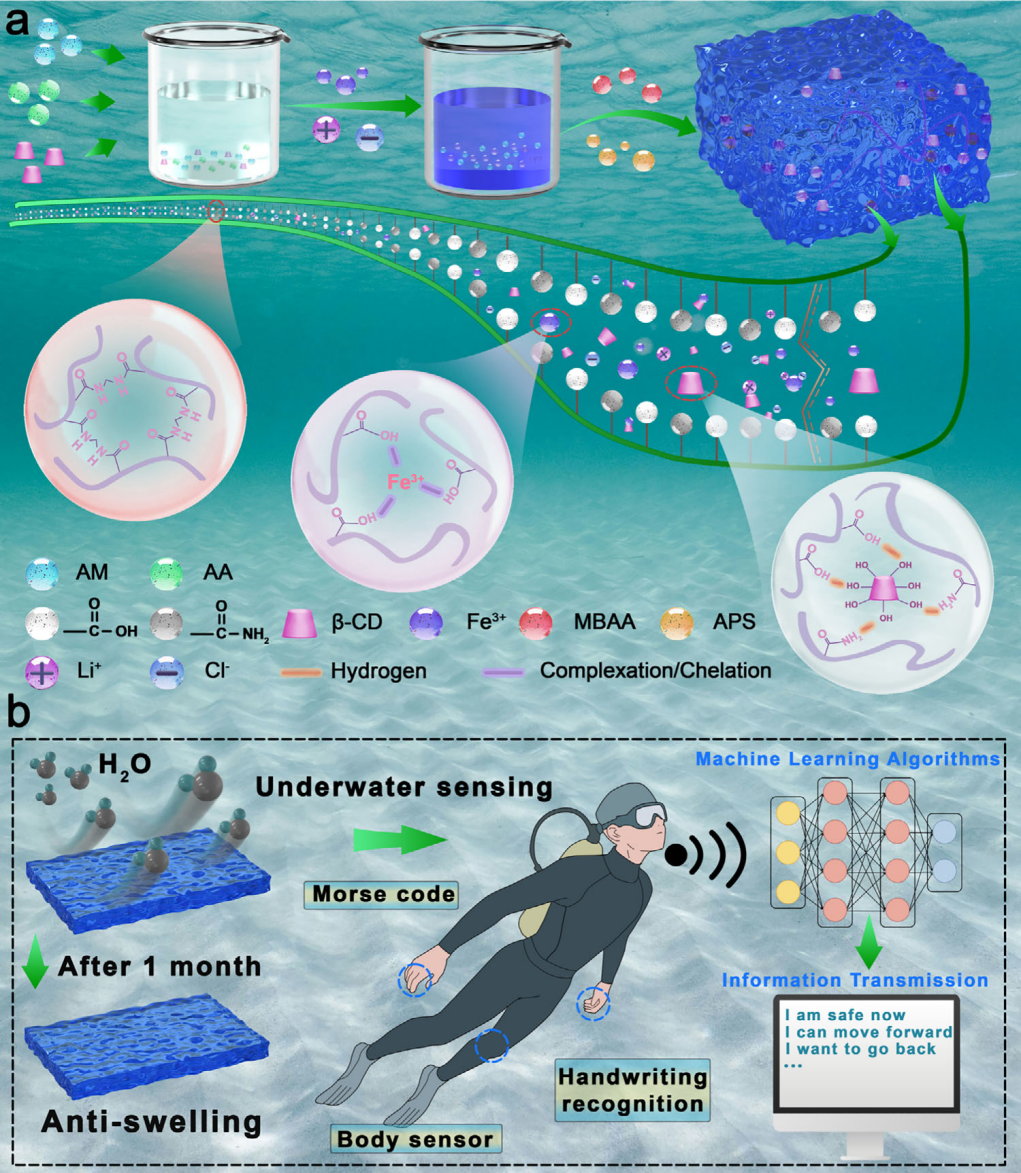
(a) Schematic synthesis of anti‐swelling MAF*_x_* D*_y_* hydrogels and (b) for underwater sensing applications combined with machine learning algorithms.

The schematic one‐pot synthesis and for underwater‐sensing applications of the MAF*_x_* D*_y_* hydrogels are shown in Figure 1a. Firstly, AM, AA, FeCl_3_, and β‐CD were dissolved in the deionized water. LiCl was used as the conductive agent, MBAA as the cross‐linking agent, and APS as the initiator were added to the solution. After a sufficiently homogeneous mixing of the components by magnetic stirring, the mixture was rapidly transferred to a polytetrafluoroethylene mold and reacted for one hour at a constant temperature of 55°C to obtain the MAF*_x_* D*_y_* hydrogels. The covalent cross‐linking reaction initiated by the free‐radical polymerization resulted in a formation of a network of polyacrylamide‐polyacrylic acid (MA) chains, which constituted the main hydrophilic framework of the MAF*_x_* D*_y_* hydrogel. β‐CD formed a large number of intermolecular hydrogen bonding via the hydroxyl groups with the carboxyl and amide groups of the MA chains [29]. These physical cross‐links act as molecular “rivets,” effectively enhancing the density of the network structure without destroying the covalent network of the main chain, thus providing the hydrogel with excellent mechanical properties and swelling resistance. The introduction of Fe^3+^ forms weak coordination complexes with the carboxyl and amide groups of the MA chain and the hydroxyl groups of β‐CD [30], which not only further enhanced the crosslinking density of the gel network but also accelerated the polymerization reaction rate (Table S3). The addition of LiCl brings a large number of free ions into the hydrogel system [31]. The multimodal underwater sensor constructed based on the MAF_0.2_D_0.3_ hydrogel is capable of monitoring underwater human joint motion and realizing the precise transmission of underwater information through Morse code (Figure 1b). Furthermore, machine learning algorithms was successfully utilized to realize high‐precision parsing of underwater handwritten trajectories and fast transmission of information through the letter recognition technology.

### 2.2 Characterizations of MAF*_x_* D*_y_* Hydrogels

**FIGURE 2:**
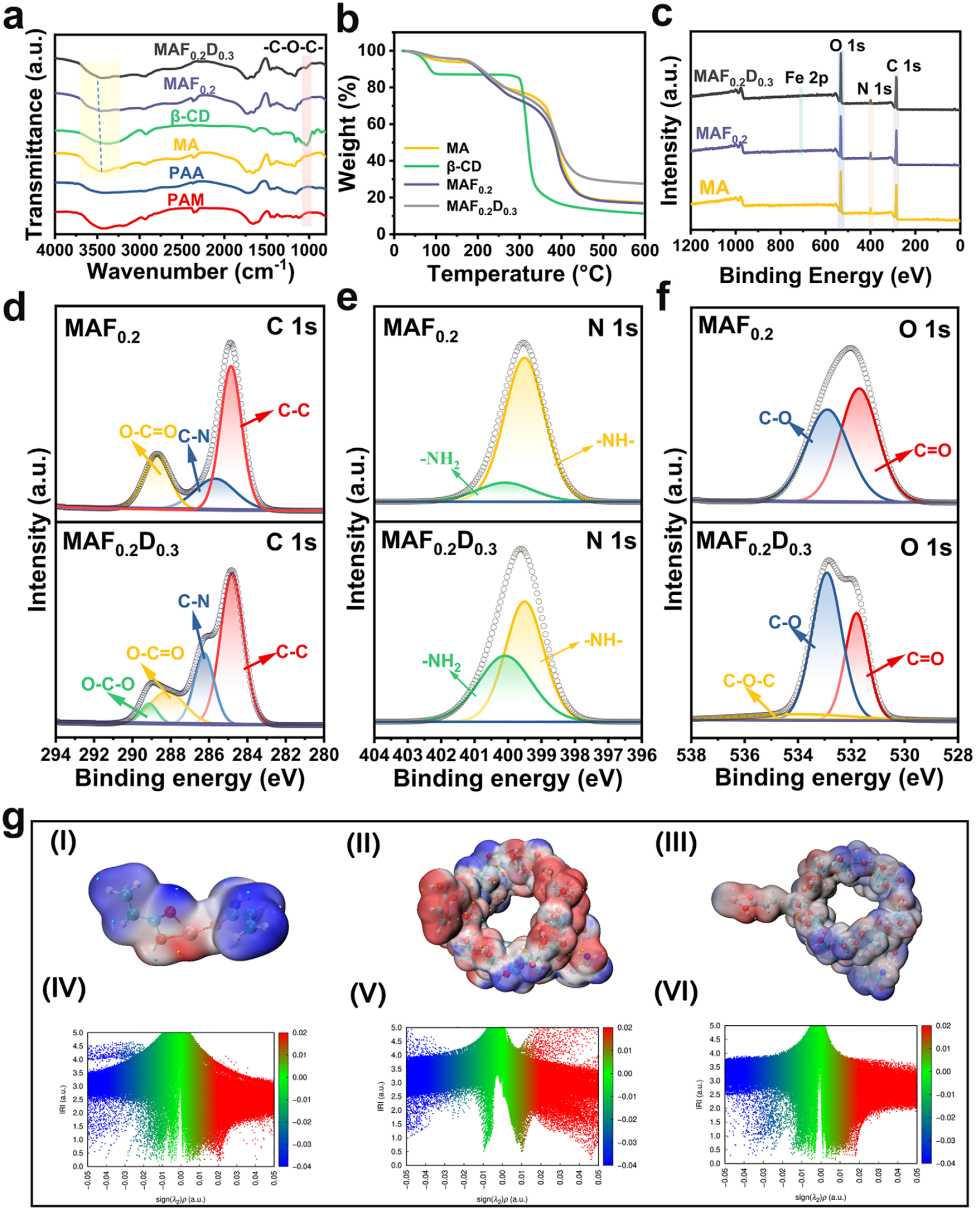
(a) FTIR spectra of MAF_0.2_D_0.3_, MAF_0.2_, β‐CD, MA, PAA, and PAM hydrogels. (b) TG curves of MAF_0.2_D_0.3_, MAF_0.2_, β‐CD and MA hydrogels. (c) XPS patterns of MAF_0.2_D_0.3_, MAF_0.2_ and MA hydrogels. High‐resolution X‐ray photoelectron spectroscopy (XPS) patterns of MAF_0.2_D_0.3_, MAF_0.2_ and MA hydrogels (d) Carbon ions C1s, (e) Nitrogen ions N 1s, and (f) Oxygen ions O 1s. (g) MAF_0.2_, MAD_0.3_ and MAF_0.2_D_0.3_ Electrostatic potential (ESP) mapping of van der Waals surfaces for (I) MAF_0.2_, (II) MAD_0.3_, (III) MAF_0.2_D_0.3_ and IGM scatter plots for (IV) MAF_0.2_, (V) MAD_0.3_ and (VI) MAF_0.2_D_0.3_.

Figure 2a demonstrates the characteristic ─C─O─C stretching vibration peak at 1082 cm^−1^, indicating that β‐CD has been successfully introduced into the MAF_0.2_D_0.3_ hydrogel network [32]. The vibration signal of ─OH (3000–3700 cm^−1^) in MAF_0.2_D_0.3_ shifts to a higher wavenumber compared with that of MA. This can be explained by the fact that the addition of β‐CD affects the number of ─C═O···H─O─ hydrogen bonds in MA [33]. The effect of β‐CD incorporation on complexation was further explored using UV–Vis spectroscopy (Figure S1). The absorption wavelength of the MAF*_x_* D*_y_* hydrogel changes significantly with increasing β‐CD content, indicating that the increase in β‐CD content leads to an increase in the number of functional groups in the system, and multiple functional groups complexed with Fe^3+^ [34]. Moreover, the addition of β‐CD significantly enhances the thermal stability of the hydrogel (Figure 2b). This is attributed to the ability of the hydroxyl groups of β‐CD to form physical cross‐links with the network structure in the MAF_0.2_ hydrogel through hydrogen bonding and van der Waals forces [9]. In order to further verify the elemental composition and surface chemical state of the MAF*_x_* D*_y_* hydrogels, the MA, MAF_0.2_, and MAF_0.2_D_0.3_ hydrogels were characterized by XPS, respectively. The XPS survey spectra show that all the three hydrogels contain the elements C, N, and O. However, the element Fe appears in the MAF_0.2_ and MAF_0.2_D_0.3_ hydrogels (Figure 2c and Figure S2) [35]. Subsequently, the C, N, and O elements were analyzed in detail. As shown in Figure 2d, the high‐resolution C1s spectrum of MAF_0.2_ can be divided into three peaks: ─C═C at 284.6 eV, ─C─N at 285.7 eV, and ─O─C═O at 288.7 eV. However, the high‐resolution C1s spectrum of MAF_0.2_D_0.3_ shows a fourth peak, ─O─C─O at 289.1 eV, which is unique to β‐CD, indicating that β‐CD was successfully introduced into the hydrogel network [36]. The high‐resolution N1s spectra shows a significant increase in the area of the ─NH_2_ peak at 400.1 eV in MAF_0.2_D_0.3_ (Figure 2e), indicating that the hydroxyl group of β‐CD forms hydrogen bonds with the amino group in the MA hydrogel. The formation of hydrogen bonds affects the distribution of electrons in the amino group, which increases the density of the electron cloud on the amino group, resulting in a stronger amino group. Similarly, high‐resolution O1s spectra show that the intensity of the ─C─O peak at 532.9 eV in the MAF_0.2_D_0.3_ hydrogel is significantly larger than that at 533.3 eV in the MAF_0.2_ hydrogel compared with the MAF_0.2_ hydrogel, which is attributed to the presence of hydroxyl functional groups in β‐CD. The ─C─O─C peak at 533.9 eV is unique to β‐CD, which proves that β‐CD forms an effective cross‐linking structure with the MAF_0.2_ hydrogel (Figure 2f) [37]. In addition, theoretical calculations were used to further investigate the effects of introducing Fe^3+^ and β‐CD on the intermolecular interaction mechanism of MA hydrogels. By analyzing the electrostatic potentials on the surfaces of MAF_0.2_, MAD_0.3_, and MAF_0.2_D_0.3_, it is found that the molecular chains of MAF_0.2_ hydrogel are strongly electronegative, and the electron cloud densities are significantly higher than those of the molecular chains of MAD_0.3_ hydrogel. When MAF_0.2_ combines with MAD_0.3_ to form MAF_0.2_D_0.3_, the distribution of molecular electron cloud density becomes more uniform (Figure 2g‐I–III), which is a phenomenon that fully proves that the electron cloud transfer occurs between the two through the chemical bond connection, which in turn enhances the intermolecular interaction [38]. To further quantify the intermolecular interactions, the study utilized an independent gradient model (IGM) was used to visualize and quantify the hydrogen bonding and electrostatic interactions in the molecules of MAF_0.2_, MAD_0.3_, and MAF_0.2_D_0.3_ (Figure 2g‐IV–VI) [39]. This is due to the large spatial site resistance brought about by the unique cyclic structure of β‐CD, such that there is an obvious repulsion between MAD_0.3_ molecules. However, this situation was disrupted by the introduction of Fe^3+^, which forms coordination bonds with the system molecules and effectively weakens the repulsive force between the polymers. Compared with MAD_0.3_ molecules, the electrostatic interactions and hydrogen bonding of MAF_0.2_D_0.3_ molecules are significantly enhanced. The introduction of Fe^3+^ not only promotes the construction of the crosslinked network structure of MA hydrogels but also strengthens the interfacial bonding within the polymer framework.

### 2.3 Mechanical Properties of MAF*_x_* D*_y_* Hydrogels

**FIGURE 3:**
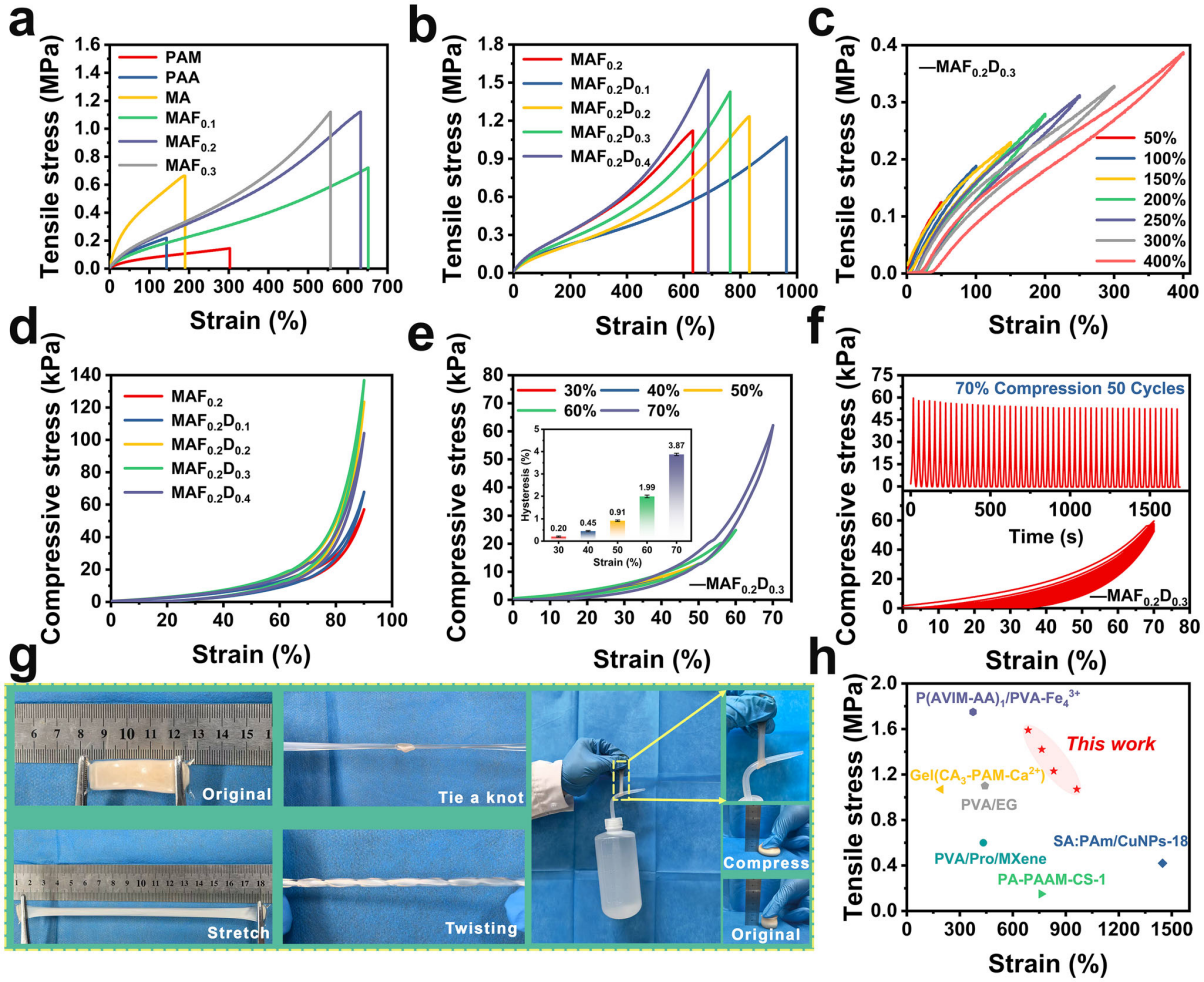
(a) Tensile stress–strain curves of PAM, PAA, and MAF*_x_* hydrogels. (b) Tensile stress–strain curve of the MAF*_x_* D*_y_* hydrogels. (c) Tensile cycling curves of MAF_0.2_D_0.3_ hydrogel at different strains (50%, 100%, 150%, 200%, 250%, 300% and 400%). (d) Compressive stress–strain curve of MAF*_x_* D*_y_* hydrogels at 90% compressive strain. (e) Corresponding energy dissipation of the MAF_0.2_D_0.3_ hydrogel at different compressive strains (*n* = 3 independent samples). (f) Loading–unloading curve of MAF_0.2_D_0.3_ hydrogel at 70% compressive strain at the 50th cycle. (g) Photographs of the MAF_0.2_D_0.3_ hydrogel under stretched, tied a knot, twisted, compressed, and under a load of 500 g. (h) Comparison of mechanical properties of MAF*_x_* D*_y_* hydrogels with previously reported hydrogels [42, 43, 44, 45, 46, 47].

The excellent mechanical properties of hydrogels make them suitable for application in wearable electronic devices. To systematically investigate the effects of different compositions on the mechanical properties and obtain optimal hydrogels suitable for wearable electronic devices, a series of tensile tests were performed on MAF*_x_* D*_y_* hydrogel samples with different compositions. As shown in Figure 3a and Figure S3, the tensile strength initially increases and then decreases, and the strain at break gradually decreases as the FeCl_3_ content increases from 0.1 to 0.3 wt%, up to 651.59% and toughness up to 3.25 MJ m^−3^. The enhanced tensile strength and toughness of the MAF*_x_* hydrogel compared to those of the MA hydrogel are attributed to the enhanced coordination between the molecular chains of Fe^3+^ and the polymer network, resulting in an increased crosslink density and enhanced tensile strength and toughness. However, with the gradual increase in Fe^3+^ content, the crosslink density increases, limiting the movement of the polymer chains and leading to a decrease in the strain at break [40]. To evaluate the effect of β‐CD on MAF*_x_* D*_y_* hydrogels, the tensile properties of β‐CD hydrogels containing 0.2 wt% of Fe^3+^ at different β‐CD ratios were compared (Figure 3b and Figure S4). As the β‐CD content increases from 0.1 to 0.4 wt%, the abundant hydroxyl groups on β‐CD form multiple hydrogen bonds and coordination interactions with the MA chains and Fe^3+^, which enhances the strength of the internal network structure and thus improves the tensile properties. However, as the content of β‐CD continues to increase, the rigidity of the hydrogel continued to increase, which affects its flexibility and ductility [41]. The strain‐at‐break and toughness of the MAF*_x_* D*_y_* hydrogels initially increases and then decreases, reaching optimum mechanical properties with a toughness of 4.52 MJ m^−3^ at a β‐CD content of 0.3 wt%. Therefore, the MAF_0.2_D_0.3_ hydrogel with a Fe^3+^ content of 0.2 wt% and a β‐CD content of 0.3 wt% exhibits the best mechanical properties and was selected for further research. Figure 3c shows the typical loading–unloading curves of the MAF_0.2_D_0.3_ hydrogel at different strains. Even when stretched to 400%, the MAF_0.2_D_0.3_ hydrogel can still return to its original state, and as the strain increases from 50% to 400%, the corresponding dissipated energy rises from 3.31 to 107.95 kJ m^−3^. This suggests that at larger strains, the MAF_0.2_D_0.3_ hydrogel can be protected by the fracture of reversible noncovalent interactions, protecting the hydrogel from permanent deformation. In addition, owing to the synergistic effect of the chemical and physical crosslinking interactions, the MAF_0.2_D_0.3_ hydrogel exhibited excellent flexibility and deformation resistance. As shown in Figure 3g, the MAF_0.2_D_0.3_ hydrogel can withstand various deformations, such as compression, stretching, knotting, twisting, and mutual pulling, and the hydrogel with a thickness of 1.5 mm and a width of 10 mm can easily withstand a load of 500 g without damage, which demonstrates that this hydrogel exhibits super toughness. Comparing the MAF*_x_* D*_y_* hydrogel with previously reported hydrogels (Figure 3h and Table S4), the superiority of the unique mechanical properties of the MAF*_x_* D_y_ hydrogel is further highlighted by comparing the key mechanical properties of tensile strength and elongation at break. The compression and recovery properties of hydrogels are crucial for their practical applications. In this study, the compressive strength and recovery properties of MAF*_x_* D*_y_* hydrogels were investigated by varying the β‐CD content. The compressive stress–strain curves in Figure 3d show that the compressive strength of the hydrogel increased and then decreased with increasing β‐CD content, as shown in Figure S5. The MAF_0.2_D_0.3_ hydrogel exhibited the highest compressive strength of 136.79 kPa at 90% strain, which was much higher than that of MAF_0.2_ (57.21 kPa). As shown in Figure 3e, MAF_0.2_D_0.3_ exhibited very low energy dissipation (3.87%) at 60% compression, and although hysteresis loops were also observed after 50 cycles, no significant degradation of performance was observed (Figure 3f), further highlighting its excellent fatigue resistance. Even after 200 loading–unloading compression cycles at 40%, MAF_0.2_D_0.3_ exhibited a stable compressive strength (Figure S6).

### 2.4 Anti‐Swelling Properties of the MAF_0.2_D_0.3_ Hydrogel

**FIGURE 4:**
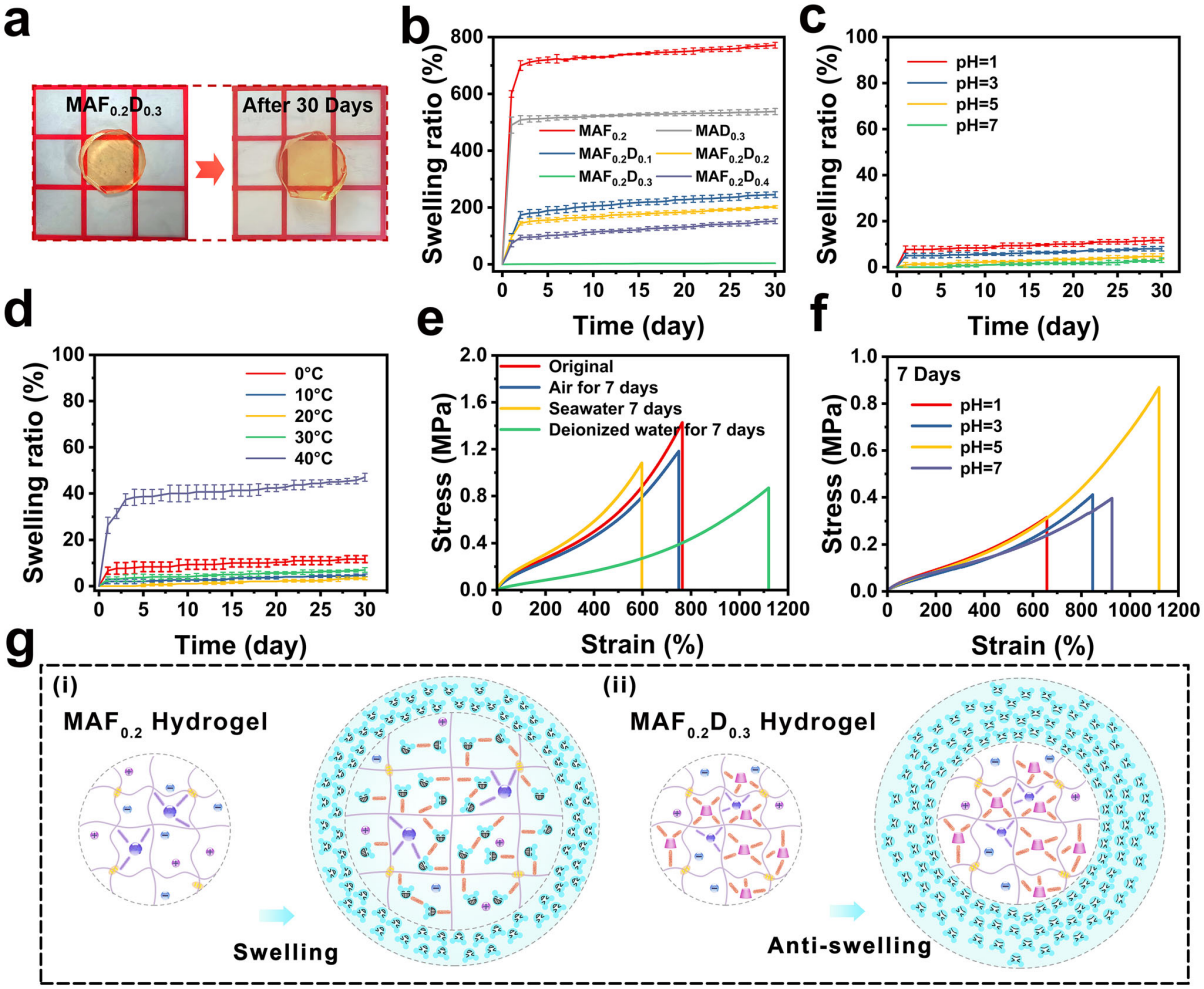
(a) Before and after swelling of the MAF_0.2_D_0.3_ hydrogel. (b) Dissolution kinetics profiles of MAF_0.2_, MAD_0.3_ and MAF*_x_* D*_y_* hydrogels in deionized water. Dissolution kinetics profiles of MAF_0.2_D_0.3_ hydrogels in (c) aqueous solutions at different pH values, and (d) deionized water at different temperatures (*n* = 3 independent samples). (e) Tensile mechanical properties of MAF_0.2_D_0.3_ hydrogel after 7 d of immersion in air, deionized water, and seawater. Tensile mechanical properties of MAF_0.2_D_0.3_ hydrogel after 7 d of immersion in aqueous solutions of different pH values. (g) Schematic representation of the swelling mechanism of the MAF_0.2_ hydrogel and the anti‐swelling mechanism of the MAF_0.2_D_0.3_ hydrogel.

The water‐absorption and swelling properties of hydrogels lead to a decrease in their structural and functional stability, and therefore the anti‐swelling property of hydrogels is critical for maintaining stable sensing performance in aqueous environments [26, 27]. As shown in Figure 4a, after the addition of β‐CD and Fe^3+^, the MAF_0.2_D_0.3_ hydrogel exhibits a significantly reduced equilibrium swelling ratio of 4%. The MAF_0.2_ hydrogel without β‐CD and the MAD_0.3_ hydrogel without Fe^3+^ swells severely in water with equilibrium swelling ratio of 760% and 526%, respectively (Figure 4b and Figure S7). Notably, the MAF_0.2_D_0.3_ hydrogel has a much lower equilibrium swelling ratio than many previously reported anti‐swelling hydrogels (Table S5). Excellent anti‐swelling properties are key to maintaining the stable electronic properties of hydrogels in underwater applications. In this study, the MAF_0.2_D_0.3_ hydrogel were immersed in methanol, ethanol, N,N‐dimethylformamide, and various other solvents (including n‐hexane, dichloromethane, dimethyl sulfoxide, acetonitrile, seawater, and acetone) for 30 d, as shown in Figure S8. The MAF_0.2_D_0.3_ hydrogel were severely swollen in N,N‐dimethylformamide and dimethyl sulfoxide. This may be due to the good polar similarity between the MA chains and N,N‐dimethylformamide and dimethyl sulfoxide, which favors the swelling of MAF_0.2_D_0.3_ hydrogels in N,N‐dimethylformamide and dimethyl sulfoxide. In addition, the hydrogels underwent slight swelling (≈4%) in methanol, ethanol, and n‐hexane and slight shrinkage in acetonitrile and acetone. Considering the complex underwater environment, aqueous solutions with different pH values (pH = 1–7) were prepared in this study to evaluate the swelling behavior of MAF_0.2_D_0.3_ hydrogels over 30 d. As shown in Figure 4c, the MAF_0.2_D_0.3_ hydrogel had stable anti‐swelling properties in aqueous solutions with pH values below 7, and swelling occurred above pH 7 because Fe^3+^ is susceptible to hydrolysis under strong alkaline conditions, which further weakened the ligand cross‐linking effect and promoted the swelling of the hydrogels. Subsequently, this paper further investigated the swelling resistance of MAF_0.2_D_0.3_ hydrogels under different temperature conditions over a 30 days period (Figure 4d), with slight swelling occurring between more than 30–40 °C, and slight swelling (5%) occurring below 30°C, further indicating that the hydrogels have an excellent performance in the complex underwater environments, further indicating that hydrogels have excellent swelling resistance. Hydrogels also require special mechanical stability for underwater application [22]. Therefore, the mechanical properties of the MAF_0.2_D_0.3_ hydrogels were evaluated after 7 d of immersion in air, seawater, and deionized water. As shown in Figure 4e, the MAF_0.2_D_0.3_ hydrogel exhibited stable mechanical property. After 7 days of immersion in ionized water, the tensile strength was slightly weakened (0.87 MPa) compared to that before immersion, but there was a significant increase in the tensile strain (1120.1%). In addition, the hydrogels showed excellent mechanical properties after 7 days of immersion in aqueous solutions with different pH values (Figure 4f), and the hydrogel tensile strains increased at pH 3–7. Meanwhile, the MAF_0.2_D_0.3_ hydrogel retained good mechanical properties after 7 days of immersion in deionized water at different temperatures (Figure S9). It can be seen that the equilibrium swelling ratio of the MAF_0.2_D_0.3_ hydrogel gradually decreases with the addition of β‐CD to the hydrogels, with the optimal addition of 0.3 wt%. This is because the addition of β‐CD introduces more hydroxyl groups, enhancing intramolecular and intermolecular physical interactions within the hydrogel. These interactions disrupt the hydrogen bonding between hydrophilic polymer chains and water molecules. This reduces the exposure of hydrophilic polymer chains to water molecules and weakens the interaction force between polymers and water molecules, thereby changing the anti‐swelling properties of the hydrogel. When the amount of β‐CD added exceeds 0.3 wt%, there is too much β‐CD inside the hydrogel, increasing the spatial site resistance between the chain segments of the gel network, leading to a decrease in the crosslinking density, a looser network structure, and an easier diffusion of the water molecules into the interior of the gel (Figure S10). This results in stronger interactions between polymer chains and water molecules, thereby increasing the hydrogel's swelling ratio in water. To further substantiate the aforementioned viewpoints, this work constructed two system models: one without β‐CD and one containing β‐CD (Figure S11). Figure S12 shows that the *E*(β‐cyclodextrin‐H_2_O) value is significantly lower than the E(H_2_O–H_2_O) value, indicating that water molecules readily form hydrogen bonds with β‐cyclodextrin. The mean square displacement (MSD) curves (Figure S13) reveal that in the system without β‐CD, the MSD curve of water molecules increases rapidly, with a diffusion coefficient of 1.73 × 10^−6^ cm^2^ per ps. Upon introducing β‐CD, the MSD curve of water molecules becomes markedly flatter, with the diffusion coefficient decreasing to 1.61×10^−6^ cm^2^ per ps, indicating that water molecule motion is significantly constrained. The synergistic interaction between hydrogen bonds formed among PMA chains, water molecules, and β‐CD effectively enhances the cohesion of the system, thereby significantly improving anti‐swelling performance. Furthermore, quantitative analysis of hydrogen bond counts (Figure S14) indicates a 15.5% increase in hydrogen bonds in the β‐CD‐containing system compared to the β‐CD‐free system. This demonstrates that the introduction of β‐cyclodextrin significantly promotes the formation of an internal hydrogen bond network within the gel, constructing a more densely cross‐linked structure at the molecular level. This enhanced hydrogen bond interaction network provides a theoretical basis for explaining the superior anti‐swelling performance exhibited by gels containing β‐CD. The swelling behavior of the MAF_0.2_ and MAF_0.2_D_0.3_ hydrogels can be explained by the following mechanism (Figure 4g). In this study, MAF_0.2_ hydrogels with absorptive swelling behavior (Figure 4g‐i) and MAF_0.2_D_0.3_ hydrogels with excellent swelling resistance (Figure 4g‐ii) were used as examples to explain the swelling behavior of hydrogels in more detail. The β‐CD‐free MAF_0.2_ hydrogel exhibits a large swelling property (760%) owing to the interactions between the hydrophilic groups of the MA chains and the water molecules, which weakens the interactions between the polymer chains and favored the penetration of water into the hydrogel network. However, significant swelling is inhibited by the significant electrostatic repulsion between the MA chains and Fe^3+^ (760%). However, the introduction of β‐CD enhances intramolecular and intermolecular interactions within the polymer chains through two mechanisms: hydrogen bonding between the hydroxyl groups of β‐CD and MA molecular chains, and coordination between β‐CD hydroxyl groups and Fe^3+^. Rheological testing further demonstrates that this dual interaction increases the crosslinking density and forms a more compact hydrogel network (Figure S15), thereby improving the hydrogels’ swelling resistance [48]. The excellent anti‐swelling and mechanical properties of the MAF_0.2_D_0.3_ hydrogel make it suitable for use as a strain sensor underwater. Figure S16 shows the variation in the relative resistance of the hydrogel with tensile strain. The results clearly show that the MAF_0.2_D_0.3_ hydrogel sensor has a significant sensitivity (GF_3_ = 3.42) and a wide sensing range (400%). As shown in Figure S17, the MAF_0.2_D_0.3_ hydrogel sensor was able to clearly and accurately identify the change in Δ*R*/*R*_0_ signal during cycling in terms of strain. The MAF_0.2_D_0.3_ hydrogel sensor exhibited superior mechanical properties compared to previously reported hydrogel sensors, which not only has excellent mechanical properties but also possesses high sensitivity and a wide sensing range, making it a reliable underwater sensor (Table S6).

### 2.5 Underwater Motion Sensors

**FIGURE 5:**
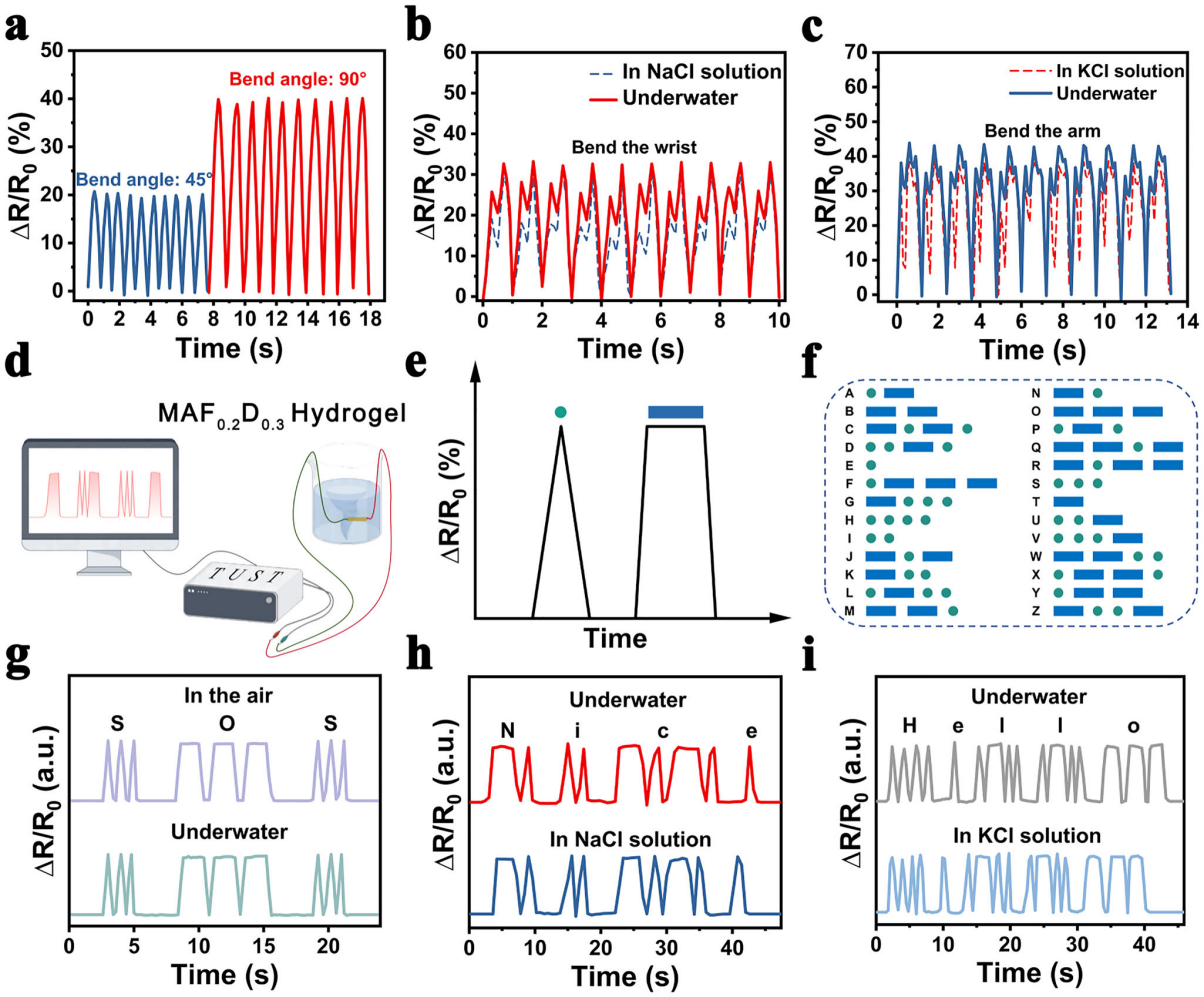
Resistance changes of the MAF_0.2_D_0.3_ hydrogel as a sensor underwater or in NaCl solution, or KCl solution for (a) finger bending, (b) wrist bending, and (c) elbow. (d) Schematic of the MAF_0.2_D_0.3_ hydrogel sensor for underwater sensing. e) Photographs of the finger stretching and bending processes. (f) Schematic diagram of the 26 English letters corresponding to Morse code. (g) Transmission of the Morse code message “SOS” by the MAF_0.2_D_0.3_ hydrogel sensor in air and deionized water. (h) Transmission of the Morse code message “Nice” by the MAF_0.2_D_0.3_ hydrogel sensor in deionized water and NaCl solution. (i) Transmission of the Morse code message “Hello” by the MAF_0.2_D_0.3_ hydrogel sensor in deionized water and KCl solution.

To further demonstrate the performance of the MAF_0.2_D_0.3_ hydrogel sensor in response to these compound strains, the MAF_0.2_D_0.3_ hydrogel sensor was affixed to different joints of the human body to accurately monitor the different motions of volunteers. As shown in Figure S18, the MAF_0.2_D_0.3_ hydrogel sensor detects the motion signals of swallowing, arm bending, and foot lifting, and it can be seen that the motion signals are arranged in a regular pattern. It is further shown that the MAF_0.2_D_0.3_ hydrogel sensor can detect not only small body movements but also large and repetitive body movements. Owing to the hydrogen bonding of β‐CD, the MAF_0.2_D_0.3_ hydrogel exhibits remarkable anti‐swelling properties, making it possible for underwater sensing applications. MAF_0.2_D_0.3_ hydrogels are attached to the fingers, wrists, and arms to investigate the sensing performance in underwater environments, Figure 5a–c, the MAF_0.2_D_0.3_ hydrogel not only maintains good signal stability underwater but also produces stable and reproducible signals in different salt solutions (i.e., NaCl and KCl solutions) with varying concentrations [49, 50]. In addition, the MAF_0.2_D_0.3_ hydrogel exhibited response characteristics highly consistent with the bending‐release motion cycle of underwater joints and maintained good structural and functional stability after prolonged underwater loading (Figure S19), indicating that the MAF_0.2_D_0.3_ hydrogel sensor holds great promise for real‐time monitoring of human motion in underwater environments. To further validate its promising application, the MAF_0.2_D_0.3_ hydrogel sensor was used to construct a real‐time underwater human motion detection system, in which the electrical signals generated by human body motion were effectively converted into Morse code, thus realizing precise information transmission from the sensor (Figure 5d). Specifically, the sharp peaks generated by short‐term bending are defined as “dots,” while the peaks with a plateau generated by long‐term bending are defined as being in a “line” state (Figure 5e). In combination with the Morse code table (Figure 5f), the combination of the letters “SOS” was successfully detected in air, while in the underwater environment, the sensor was able to successfully detect the letters “SOS” (Figure 5g). To verify the information transfer capability of the MAF_0.3_ hydrogel sensor under different environmental conditions, we further explored its performance in special solutions. As shown in Figure 5h,i, hydrogel sensors stably transmitted information and successfully detected the combination of the words “Nice” and “Hello” despite the presence of different salt solutions (NaCl and KCl solutions). This result further demonstrates the robustness and reliability of the MAF_0.2_D_0.3_ hydrogel sensor in different underwater environments, particularly complex ones.

### 2.6 Underwater Handwriting Recognition

**FIGURE 6:**
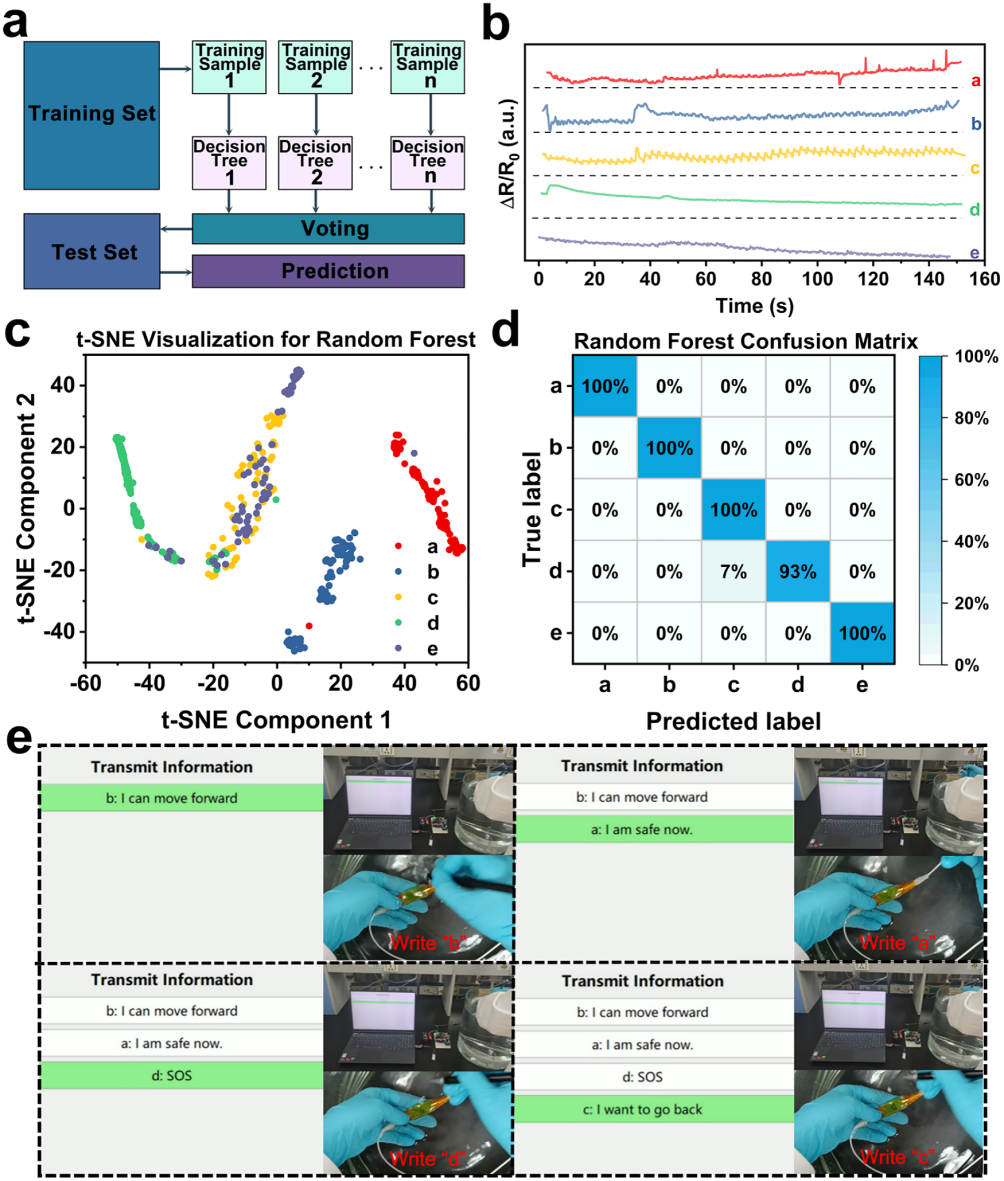
(a) Logic diagram of data classification using the Random Forest model. (b) Resistance value changes by writing “a,” “b,” “c,” “d,” “e” for 100 times. (c) t‐distributed Stochastic Neighbor embedding (t‐SNE) visualization diagram. (d) Confusion matrix diagram of the recognition results of five letters by the random forest model. (e) Underwater intelligent alarm system assembled by hydrogels and its output statements in different states.

To further validate the capability of this MAF_0.2_D_0.3_ hydrogel sensor in multimodal recognition, a set of processes were designed (Figure S20) in which the user wrote different letters underwater for information transmission. The hydrogel sensor captures these changes of the pressure in real time and generates microcurrent signals, which are transmitted to the computer through a handwriting recognition system and are subsequently classified using a machine learning algorithm to produce the final results. The key encapsulation technique (Figure S20‐i) plays a crucial role in maintaining the stability of the hydrogel underwater, effectively suppressing ion leakage from the hydrogel sensor (Table S7). Benefiting from the hydrogel's excellent adhesion properties (Figure S21), it maintains a robust bond with the encapsulation material even after prolonged underwater immersion (Figure S22), thereby ensuring the sensor's long‐term stable operation in aquatic environments. As shown in Figure S20‐ii, to ensure user comfort and build a self‐sufficient system, a hydrogel‐based intelligent writing recognition system was constructed by combining signal processing circuits and a host‐computer interface, which was used to collect and process the signals generated by the sensor and send them to the computer, Figure S20‐iii. Machine learning is a data‐driven approach for empirically approximating unknown models. Decision tree algorithms are preferred for their classification capabilities, where multiple decision tree classifiers are generated to create a random forest model, each decision tree makes predictions independently, and ultimately votes to produce a final classification prediction (Figure 6a) [51]. Therefore, a high‐performance random forest algorithm was used for the signal classification. To evaluate the model's performance and stability under simulated practical underwater conditions, the sensing data of the MAF_0.2_D_0.3_ hydrogel sensor were investigated by writing five representative letters: “a,” “b,” “c,” “d,” and “e.” To account for potential underwater disturbances such as water flow and pressure fluctuations, a data augmentation strategy was employed during the training phase. Specifically, Gaussian white noise of varying variances and baseline drift operators were superimposed on the raw micro‐current signals to construct a more challenging and robust dataset. The results in Figure 6b indicate that the sensor outputs stable and highly repeatable signals during underwater deformation cycles. The augmented dataset samples are randomly partitioned into two disjoint subsets at a ratio of 4:1 (the training set and the test set). These training samples were then used to construct the random forest prediction model. In addition, the t‐distributed stochastic neighborhood embedding (t‐SNE) algorithm was employed to downscale the high‐dimensional features extracted from the encoder to analyze the recognition of these actions at greater depths (Figure 6c). The samples of the five different states form distinct clusters in a feature space where the feature points of each category are accurately mapped to the labels of these classifications, thereby highlighting the robustness of the model in this scenario. To validate the performance of the model, a confusion matrix for recognizing the five letter categories of the test was generated after training, and the overall recognition accuracy reached 98.60% (Figure 6d). A prototype system was further developed for underwater handwriting sensing and critical state monitoring based on an MAF_0.2_D_0.3_ hydrogel sensor. Specifically, to balance communication efficiency with the physiological constraints of underwater operators in emergency scenarios, the communication protocol was intentionally customized and simplified. Instead of full‐alphabet input, a discrete command‐mapping strategy was adopted to achieve rapid, silent information transfer between the underwater environment and the shore. As a demonstration (Figure 6e and Video S1), a finger generates distinct micro‐current signals during rapid writing. Five essential transmission statements were predefined: writing the letter “b” triggers the “I can move forward” message, while “a,” “d,” and “c” correspond to “I am safe now,” “SOS,” and “I want to go back,” respectively. Although this proof‐of‐concept focuses on core emergency commands, the distinct clustering observed in t‐SNE analysis (Figure 6c) underscores the system's capacity for expanded vocabularies. Furthermore, by incorporating transfer learning techniques, the model can be rapidly recalibrated with minimal samples to adapt to the diverse handwriting styles of different users, ensuring robust cross‐user generalization. Overall, this study successfully constructed and demonstrated an underwater safety monitoring and auxiliary communication platform with practical application prospects, which provides a new technical method for the safety of underwater operators.

## 3 Conclusion

In summary, the MAF*_x_* D*_y_* hydrogels were successfully constructed by introducing Fe^3+^ and β‐CD into the acrylamide‐acrylic acid copolymer (MA) network via the one‐pot method. The introduction of Fe^3+^ and β‐CD significantly enhanced the mechanical properties of the hydrogels, in which the MAF*_x_* D*_y_* hydrogel exhibited excellent mechanical tensile properties, with a strain at break of up to 962.33% and fracture toughness. The maximum fracture strain was 4.52 MJ m^−3^. The MAF_0.2_D_0.3_ hydrogel exhibited excellent swelling resistance (4%) after 30 d of underwater immersion and maintained an intact network structure and stable mechanical properties. The flexible sensor developed based on the MAF_0.2_D_0.3_ hydrogel exhibited high sensitivity (strain factor GF = 3.42) and a wide sensing range (0–400%), and maintained reliable sensing performance after immersion at different temperatures (0–40°C) or different pH (1–7). Notably, the hydrogel can not only be directly applied to underwater motion monitoring but realize the precise transmission of information through Morse code encoding. Further, combined with machine learning algorithms, the intelligent recognition of underwater handwritten characters was successfully realized with a recognition accuracy of 98.6%. This study breaks through the bottleneck that it is difficult to balance the mechanical properties and environmental adaptability of traditional hydrogels and provides a new paradigm for the design of intelligent soft materials with both high‐strength and swelling‐resistant properties. The developed MAF_0.2_D_0.3_ hydrogel sensor shows broad application prospects in underwater wearable health devices, and is expected to promote the practical application of flexible electronic devices in complex underwater environments.

## 4 Experimental Section

### 4.1 Materials

Acrylamide (99%, AM), acrylic acid (99.5%, AA), lithium chloride (99%, LiCl), and ammonium persulfate (>98%, APS) were purchased from Aladdin Co., Ltd. in China, β‐cyclodextrin (97%, β‐CD), ferric chloride (98%, FeCl_3_) were purchased from Shanghai Maclean's Biochemistry & Technology Co. Ltd, N,N'‐methylenebisacrylamide (97%, MBAA) was purchased from Shanghai BiDe Pharmaceutical Technology Co. Deionized water (DI) was produced in the laboratory. All chemicals were used directly without further purification.

### 4.2 Preparation of MAF*_x_* D*_y_* Hydrogels

MAF*_x_* D*_y_* hydrogels were prepared using a one‐pot method, where M, A, F, and D stand for acrylamide, acrylic acid, Fe^3+^, and β‐CD, respectively (where *x* and *y* represent the added mass fractions of FeCl_3_ and β‐CD, respectively). First, 1.5 g of AM, 3 ml of AA, and an appropriate amount of β‐CD (0–0.4 wt%) were added to a beaker containing 10 mL of deionized water, and the mixture was stirred for 24 h until fully dissolved. Then, 8 mg of MBAA, 20 mg of APS, 0.1 g of LiCl, and appropriate FeCl_3_ (0–0.3 wt%) were sequentially added to the above solution, and stirring was continued for 5 min. The above mixed solution was injected into a homemade polytetrafluoroethylene mold and kept at 55°C for 1 h to obtain hydrogels with cross‐linked networks. The content of each component is shown in Table S1.

### 4.3 Characterizations

The top view of the freeze‐dried samples was observed using a field emission high‐resolution scanning electron microscope (FE‐SEM) model Apreo from FEI (USA). The samples were prepared by mixing potassium bromide and different components of the gel powder at a ratio of approximately 100:1 and then pressed into tablets. The hydrogels were scanned using a VECTOR 22 infrared spectrometer (Bruker, Germany) at a resolution of 4 px^−1^ in a scanning interval of 400–4000 cm^−1^. Hydrogel solutions of different compositions were loaded into quartz cuvettes against a background of deionized water, and the UV spectral absorption was determined using an Agilent Cary 100 UV–Vis spectrophotometer in the wavelength range of 350–600 nm. The crystal structure information of the hydrogels was obtained using an X‐ray diffractometer (Malvern Panalytical‐Empyren). The surface elemental compositions of the hydrogels were analyzed using X‐ray photoelectron spectroscopy (AXIS Supra+). The hydrogels were subjected to thermogravimetric analysis (TGA) using a simultaneous thermal analyzer (SDT‐Q600) under a nitrogen atmosphere with a scanning temperature range of 0–600°C and a heating rate of 10°C min^−1^.

### 4.4 Mechanical Tests

Tensile stress–strain tests were performed on the cylindrical hydrogel (40 × 10 × 1.5 mm) using an electronic universal testing machine (ZQ‐990LB) at a speed of 80 mm min^−1^. The toughness of the hydrogel was calculated from the area of the tensile strain‐stress curve.

Compression tests were carried out on cylindrical hydrogels (about 15 mm in diameter and 20 mm in height) using a compression property tester (WTS – 1010W) at a speed of 1 mm min^−1^. The energy consumption of the hydrogels was calculated from the area under the compressive stress–strain curve.

### 4.5 Anti‐Swelling Properties Tests

The swelling of the hydrogels was detected by immersing rectangular samples (10 mm × 40 mm × 2 mm) in the deionized water and recording the weights of the samples after different immersion times. MAF_0.2_D_0.3_ hydrogels were also immersed in different solvents separately to detect the swelling of the hydrogels. To further explore the environmental stability of MAF_0.2_D_0.3_ hydrogels, parallel specimens were immersed in solutions with different pH values and at different temperatures. The specific test conditions are presented in Table S2. The swelling rate (SR) was calculated according to Eq. (1):

(1) $$SR=\frac{W_{original}-W_{swelling}}{W_{original}}\times 100\%$$

where *W*_swelling_ (g) represents the weight of the hydrogel at a certain time period during the soaking process, and *W*_original_ (g) is the original weight of the hydrogel.

### 4.6 Electromechanical Tests

The sensing performance of hydrogel sensors was evaluated using an electrochemical workstation (Shanghai Chenhua CHI 760E), using the same strips as those used in the mechanical testing. The sensitivity, the gauge factor (GF), is defined by Eq. (2):

(2) $$GF=\frac{\Delta R/R_{0}}{\xi }=\frac{\left(R-R_{0}\right)/R_{0}}{\xi }$$

where *ξ* represents the deformation magnitude of the hydrogel strip, *R* denotes the real‐time resistance, and *R_0_* indicates the initial resistance.

### 4.7 Underwater Sensing Performance Tests

The MAF_0.2_D_0.3_ hydrogel sample was clamped between ethylene vinyl acetate foam and Cu network, encapsulated with PI film on the outside to form a sensor, and copper wires were connected at both ends of the sensor. To construct a reusable writing interface, a PET flexible contact layer was added to the sensor surface, which could be physically peeled off for nondestructive replacement. Subsequently, the assembled sensors were mounted on the test site of the volunteer's body, and the motion signals were detected. It was confirmed that informed written consent from all participants was obtained prior to the experiments with sensors and wearable technologies.

### 4.8 Simulation Methods

Quantum chemical calculations were performed using the Gaussian 09 program [52]. The initial geometries and electronic wavefunctions were obtained at the M06‐2X/def2‐SVP level. For the weak interactions presumed to exist in the ordered structures of the crystalline materials of the present study, wavefunction analyses of Mayer bond orders and interaction regional indicator IRI [53] isosurfaces were created using the Multiwfn code [54] combined with the visual molecular dynamic (VMD) program [55]. IRI can reveal chemical bonding and weak interaction regions vividly, where the blue regions indicate attractive interactions, green region indicates van der Waals interactions, and the red regions indicate repulsive interactions.

Molecular dynamics (MD) simulations and binding energy (Eint) calculations were performed using the Forcite module in Materials Studio 2020 software. The COMPASS II force field was first employed to describe interactions between molecular segments, within molecules, and between molecules. Subsequently, two system models were constructed based on the actual usage ratio: one without β‐CD and one containing β‐CD, with an initial density set at 1.2 g cm^−3^. Subsequently, all systems underwent energy minimization to eliminate any unreasonable contacts in the initial configuration. Finally, mechanical relaxation and orientation simulations were performed. Upon reaching thermodynamic equilibrium, 100 ps of NVT ensemble simulations were conducted to obtain stable microstructures. The simulation temperature was set at 498.15 K (200°C).

### 4.9 Machine Learning

To test the application of the hydrogel in underwater handwriting recognition, this experiment was carried out in the Python3.12 environment for the implementation of machine learning models, and the random forest model in the Scikit‐learn library was chosen for the construction of the classification prediction model [9]. In order to realize the collection and processing of signals generated by the hydrogel and send them to the computer, the HC‐06 data receiving module, HC‐05 data transmitting module, voltage regulator, and Arduino microcontroller self‐assembled data transmission module were used for the construction of hardware.

## Conflicts of Interest

The authors declare no conflict of interest.

## Supporting information




**Supporting File**: advs74916‐sup‐0001‐SuppMat.docx.

**Supporting Video**: advs74916‐sup‐0002‐VideoS1.mp4.

## Data Availability

The data that support the findings of this study are available from the corresponding author upon reasonable request.
