# Supplementary material for: The Synergism of β‐Cyclodextrin and Fe3+ Enabled Anti‐Swelling Ion‐Conductive Hydrogels for Multimodal Underwater Sensing Aided by Machine Learning Algorithms
Source: Adv Sci (Weinh). 2026 Mar 28;13(31):e74916. doi: 10.1002/advs.74916 (PMC13587967; doi:10.1002/advs.74916)
Supplement: Supplementary file 1 [file ADVS-13-e74916-s002.docx]

Supporting Information

**The synergism of β-cyclodextrin and Fe^3+^ enabled anti-swelling ion-conductive hydrogels for multimodal underwater sensing aided by machine learning algorithms**

*Hao Dong, Chuanliu Liu, Yiming Luo, Dongyu Si, Menghan Zhao, Fangyu Zhao, Yixue Zhang, Wei Zeng, Eman Ramadan Elsharkawy, Terence Xiaoteng Liu*, Zeinhom M. El-Bahy, Hua Hou, Zhanhu Guo*, Huige Wei****

Hao Dong, Chuanliu Liu, Yiming Luo, Dongyu Si, Menghan Zhao, Fangyu Zhao, Yixue Zhang, Wei Zeng, Huige Wei

State Key Laboratory of Bio-based Fiber Materials, Tianjin Key Laboratory of Brine Chemical Engineering and Resource Eco-utilization, College of Chemical Engineering and Materials Science, Tianjin University of Science and Technology, Tianjin, 300457, China

Email: [huigewei@tust.edu.cn](mailto:huigewei@tust.edu.cn)

Eman Ramadan Elsharkawy

Center for Scientific Research and Entrepreneurship, Northern Border University, Arar 73213, Saudi Arabia

Terence Xiaoteng Liu

Faculty of Engineering and Environment, Northumbria University, Newcastle Upon Tyne NE1 8ST, UK

E-mail: terence.liu@northumbria.ac.uk

Zeinhom M. El-Bahy

Department of Chemistry, Faculty of Science, Al-Azhar University, Nasr City 11884, Cairo, Egypt

Hua Hou

College of Materials Science and Engineering, Taiyuan University of Science and Technology, Taiyuan, 030024, China

Zhanhu Guo

Department of Mechanical and Construction Engineering, Faculty of Engineering and Environment, Northumbria University, Newcastle upon Tyne, NE1 8ST, UK

Email: [zhanhu.guo@northumbria.ac.uk](mailto:zhanhu.guo@northumbria.ac.uk)

**Table of Contents**

**Supplementary Figures**

**Figure S1.** UV-Vis spectra of MAF*_x_*D*_y_*.

**Figure S2.** XPS spectra of Fe elements in MAF_0.2_D_0.3_ hydrogel.

**Figure S3.** Toughness and strain at break of PAM, PAA and MAF*_x_* hydrogels.

**Figure S4.** Toughness and strain at the break of the MAF*_x_*D*_y_* hydrogel.

**Figure S5.** Compressive strength and energy loss of MAF*_x_*D*_y_* hydrogels at 90% compressive strain.

**Figure S6.** Loading-unloading curve of MAF_0.2_D_0.3_ hydrogel at 40 % compressive strain at the 200th cycle.

**Figure S7.** Before and after swelling of the MAF*_x_*D*_y_* hydrogel.

**Figure S8.** Swelling kinetics curves of MAF_0.2_D_0.3_ hydrogel in different solution media.

**Figure S9.** Tensile mechanical properties of MAF_0.2_D_0.3_ hydrogels after 7 days of immersion in aqueous solutions at different temperatures.

**Figure S10.** SEM spectra before and after swelling of MAF_0.2_D_0.3_, MAF_0.2_ and MAD_0.3_.

**Figure S11.** (a) Molecular dynamics snapshot of the system without β-CD, (b) Molecular dynamics snapshot of the system with β-CD.

**Figure S12.** The bond energy between water molecules and the bond energy between β-CD and water molecules.

**Figure S13.** The mean square displacement (MSD) of water molecules in systems containing and not containing β-CD.

**Figure S14.** Number of hydrogen bonds in systems containing β-CD and systems without β-CD.

**Figure S15.** Rheological testing of MAF_0.2_D_0.3_, MAF_0.2_, MAD_0.3_, and MA.

**Figure S16.** GF under different stretching (0-400%) conditions in air.

**Figure S17.** Relative resistance changes under different strain (50-400%) conditions.

**Figure S18.** Resistance changes of the MAF_0.2_D_0.3_ hydrogel as a sensor for limb movement detection for swallowing, finger bending and leg lifting

**Figure S19.** Resistance change of MAF_0.2_D_0.3_ hydrogel sensor after 5000 cycles of bending underwater

**Figure S20.** The process from underwater handwriting to transmitting the machine learning model to the final display of results.

**Figure S21.** Adhesion strength of the MAF_0.2_D_0.3_ hydrogel on different substrates (skin, rubber, PET).

**Figure S22.** Adhesion strength of the MAF_0.2_D_0.3_ hydrogel and PET before and after 15 days of underwater immersion

**Supplementary Tables**

**Table S1.** Composition and content of different hydrogels.

**Table S2.** Specific conditions for soaking hydrogels at different temperatures or pH levels.

**Table S3.** Comparison of polymerization time and polymerization temperature of hydrogel in other reported literature.

**Table S4.** Comparison of mechanical properties of hydrogel in other reported literature.

**Table S5.** Comparison of equilibrium swelling rate (underwater) of anti-swelling hydrogel in other reported literature.

**Table S6.** Comparison of sensing performance of hydrogel sensors from other reported literature.

**Table S7.** ICP-MS measurement of lithium and iron ion content in 15-day immersion solution.

**Supplementary Video**

**Video S1.** Hydrogel sensors perform handwriting recognition underwater in random order.

**Supplementary Figures**

**UV-Vis analysis**

**Figure S1.** UV-Vis spectra of MAF*_x_*D*_y_*.

XPS plots of MAF_0.2_D_0.3_ hydrogels

**Figure S2.** XPS spectra of Fe elements in MAF_0.2_D_0.3_ hydrogel.

**Figure S3.** Toughness and strain at break of PAM, PAA and MAF*_x_* hydrogels.

**Figure S4.** Toughness and strain at the break of the MAF*_x_*D*_y_* hydrogel.

**Figure S5.** Compressive strength and energy loss of MAF*_x_*D*_y_* hydrogels at 90% compressive strain.

**Figure S6.** Loading-unloading curve of MAF_0.2_D_0.3_ hydrogel at 40 % compressive strain at the 200th cycle.

**Figure S7.** Before and after swelling of the MAF*_x_*D*_y_* hydrogel.

**Figure S8.** Swelling kinetics curves of MAF_0.2_D_0.3_ hydrogel in different solution media.

**Figure S9.** Tensile mechanical properties of MAF_0.2_D_0.3_ hydrogels after 7 days of immersion in aqueous solutions at different temperatures.

**Figure S10.** SEM spectra before and after swelling of MAF_0.2_D_0.3_, MAF_0.2_ and MAD_0.3_.

**Figure S11.** (a) Molecular dynamics snapshot of the system without β-CD, (b) Molecular dynamics snapshot of the system with β-CD

**Figure S12.** The bond energy between water molecules and the bond energy between β-CD and water molecules.

**Figure S13.** The mean square displacement (MSD) of water molecules in systems containing and not containing β-CD.

**Figure S14.** Number of hydrogen bonds in systems containing β-CD and systems without β-CD.

**Figure S15.** Rheological testing of MAF_0.2_D_0.3_, MAF_0.2_, MAD_0.3_, and MA.

**Figure S16.** GF under different stretching (0-400%) conditions in air.

**Figure S17.** Relative resistance changes under different strain (50-400%) conditions.

**Figure S18.** Resistance changes of the MAF_0.2_D_0.3_ hydrogel as a sensor for limb movement detection for (a) swallowing, (b) bending the arm, and (c) bending the leg.

**Figure S19.** Resistance change of MAF_0.2_D_0.3_ hydrogel sensor after 5000 cycles of bending underwater

**Figure S20.** The process from underwater handwriting to transmitting the machine learning model to the final display of results.

**Figure S21.** Adhesion strength of the MAF_0.2_D_0.3_ hydrogel on different substrates (skin, rubber, PET).

**Figure S22.** Adhesion strength of the MAF_0.2_D_0.3_ hydrogel and PET before and after 15 days of underwater immersion

**Supplementary Tables**

**Table S1.** Composition and content of different hydrogels.

| Hydrogels | AM  (g) | AA  (ml) | β-CD  (wt%) | FeCl_3_  (wt%) | H_2_O  (ml) | MBAA  (mg) | APS  (mg) | LiCl  (g) |
| --- | --- | --- | --- | --- | --- | --- | --- | --- |
| PAM | 1.5 | 0 | 0 | 0 | 10 | 8 | 20 | 0.1 |
| PAA | 0 | 3 | 0 | 0 | 10 | 8 | 20 | 0.1 |
| MA | 1.5 | 3 | 0 | 0 | 10 | 8 | 20 | 0.1 |
| MAF_0.1_ | 1.5 | 3 | 0 | 0.1 | 10 | 8 | 20 | 0.1 |
| MAF_0.2_ | 1.5 | 3 | 0 | 0.2 | 10 | 8 | 20 | 0.1 |
| MAF_0.3_ | 1.5 | 3 | 0 | 0.3 | 10 | 8 | 20 | 0.1 |
| MAD_0.3_ | 1.5 | 3 | 0.3 | 0 | 10 | 8 | 20 | 0.1 |
| MAF_0.2_D_0.1_ | 1.5 | 3 | 0.1 | 0.2 | 10 | 8 | 20 | 0.1 |
| MAF_0.2_D_0.2_ | 1.5 | 3 | 0.2 | 0.2 | 10 | 8 | 20 | 0.1 |
| MAF_0.2_D_0.3_ | 1.5 | 3 | 0.3 | 0.2 | 10 | 8 | 20 | 0.1 |
| MAF_0.2_D_0.4_ | 1.5 | 3 | 0.4 | 0.2 | 10 | 8 | 20 | 0.1 |

**Table S2.** Specific conditions for soaking hydrogels at different temperatures or pH levels.

| **Figure** | **pH** | **Temperature** | **Solvent** |
| --- | --- | --- | --- |
| **Figure 4a/S8** | 5 | 20 °C | Deionized Water |
| **Figure 4e** | / | 20 °C | Air |
|  | 5 | 20 °C | Seawater |
|  | 5 | 20 °C | Deionized Water |
| **Figure 4b/f/h** | 1 | 20 °C | Deionized Water |
|  | 3 | 20 °C | Deionized Water |
|  | 5 | 20 °C | Deionized Water |
|  | 7 | 20 °C | Deionized Water |
|  | 9 | 20 °C | Deionized Water |
| **Figure 4c/g/i** | 5 | 0 °C | Deionized Water |
|  | 5 | 10 °C | Deionized Water |
|  | 5 | 20 °C | Deionized Water |
|  | 5 | 30 °C | Deionized Water |
|  | 5 | 40 °C | Deionized Water |

**Table S3.** Comparison of polymerization time and polymerization temperature of hydrogel in other reported literature.

| **Hydrogels** | **Polymerization time** | **Polymerization temperature** | **Complexity of preparation** | **Functional integration** | **Ref.** |
| --- | --- | --- | --- | --- | --- |
| **MAF_0.2_D_0.3_** | **1 h** | **55 °C** | **one-pot solution** | **Strain sensing/ Morse code communication/ Machine learning recognition** | **This work** |
| PAM/CMC/PEG/Ag-PDA@SiO | 4 h | 60 °C | one-pot solution | Strain sensing/ Morse code communication | [1] |
| PACCMZ | 2 h | 60 °C | multistep approach | Strain sensing | [2] |
| P(AM-co-AA)/BC/TA/LiCl | 2 h | 60 °C | one-pot solution | Strain sensing | [3] |
| PAB | 24 h | -20 °C | multistep approach | Strain sensing/ Machine learning recognition | [4] |
| PAM/CMC/DA/MXene | 3 h | 60 °C | one-pot solution | Strain sensing | [5] |

**Table S4.** Comparison of mechanical properties of hydrogel in other reported literature.

| **Hydrogels** | **Strain (%)** | **Stress (MPa)** | **Ref.** |
| --- | --- | --- | --- |
| **MAF_0.2_D_0.3_** | **764.13** | **1.42** | **This work** |
| SA:PAm/CuNPs-18 | 1448 | 0.42 | [6] |
| GelCA_3_-PAM-Ca^2+^ | 192 | 1.07 | [7] |
| PA-PAAM-CS-1 | 760.9 | 0.15 | [8] |
| P(AVIM-AA)_1_/PVA-Fe_4_^3+^ | 375 | 1.75 | [9] |
| PVA/Pro/MXene | 433 | 0.6 | [10] |
| PVA/EG | 442.3 | 1.1 | [11] |

**Table S5.** Comparison of equilibrium swelling rate (underwater) of anti-swelling hydrogel in other reported literature.

| **Hydrogels** | **Equilibrium swelling rate (%)** | **Ref.** |
| --- | --- | --- |
| **MAF_0.2_D_0.3_** | **4** | **This work** |
| PAM-ALG-PPy | ~ 100 | [12] |
| WCH | 42 | [13] |
| PVA/NaCl/Amy_4wt%_ | 28.5 | [14] |
| Gel/PSAA-Al^3+^ | 32 | [15] |
| P(SBMA-co-HEMA)/glycerol | 217 | [16] |
| P(AA-LMA)_CTAB_-Zr^4+^-Eq | 11.82 | [17] |

**Table S6.** Comparison of sensing performance of hydrogel sensors from other reported literature.

| **Hydrogel Type** | **Gauge Factor (GF)** | **Underwater sensing** | **Ref.** |
| --- | --- | --- | --- |
| **MAF_0.2_D_0.3_** | **GF_1_ = 1.87 (0-30%)**  **GF_2_ = 2.42 (30-100%)**  **GF_3_ = 3.42 (100-400%)** | **Yes** | **This work** |
| Gel-OCMC-AMS | GF_1_ = 0.64 (10-50%)  GF_2_ = 0.86 (50-100%)  GF_3_ = 0.18 (100-150%)  GF_4_ = 0.44  (150-200%) | / | [18] |
| Gel-Agar-SA-LiCl | GF_1_ = 0.47 (0-20 %)  GF_2_ = 0.16 (20-80%) | / | [19] |
| Gel/P(AM-co-AMPS) | GF_1_ = 2.58  (0–100%)  GF_2_ = 5.32  (100–500%) | / | [20] |
| PTPAVI-Al^3+^ | GF_1_ = 0.83 (0-200%)  GF_2_ = 1.44 (200-500%)  GF_3_ = 2.01 (500-800%) | Yes | [21] |
| Gel/PS_4_A_6_A-3Al^3+^ | GF_1_ = 0.83 (0-200%)  GF_2_ = 1.29 (200-600%)  GF_3_ = 1.61 (600-1050%) | Yes | [15] |

**Table S7.** ICP-MS measurement of lithium and iron ion content in 15-day immersion solution.

| **Sampling Volume V (mL)** | **Fixed Volume V_0_ (mL)** | **Test Element** | **Test Solution Element Concentration C_0_ (µg/L)** | **Dilution factor f** | **Element Concentration C_1_ (μg/L) in the Original Digestion Solution** | **Elemental Content in Sample C_x_ (μg/L)** |
| --- | --- | --- | --- | --- | --- | --- |
| / | / | Li | 0.049 | 1 | 0.049 | 0.049 |
| / | / | Fe | Not detected | 1 | Not detected | Not detected |

**References**

[1] X. Ji, Z. Yu, M. Nan, R. Ge, Y. Gao, H. Zhang, Z. Li, L. Wang, Int. J. Biol. Macromol. **2025**, 315, 144405.

[2] H. Sima, B. Liu, X. Shi, C. Zhang, Carbohydr. Polym. **2025**, 123825.

[3] H. Zhang, D. Zhang, H. Cai, Y. Ma, K. Li, P. Zhang, Y. Guo, Carbohydr. Polym. **2025**, 360, 123601.

[4] Y. Cheng, S. Fu, K. Jin, Y. Liu, J. Ma, D. Li, Q. Lyu, Y. Li, X. Jiang, R. Liu, Y. Tian, Chem. Eng. J. **2024**, 498, 155475.

[5] P. Zhang, W. Wang, Y. Ma, H. Zhang, D. Zhou, X. Ji, W. Liu, Y. Liu, D. Zhang, Chem. Eng. J. **2024**, 499, 156173.

[6] U. Muhammad, X. Cao, T. Zhang, W. Ji, R. Lv, J. Chen, Y. Wei, Int. J. Biol. Macromol. **2025**, 311, 143734.

[7] X. Gong, Y. Luo, C. Zhang, J. Xie, Y. Wang, Chem. Eng. J. **2025**, 510, 161610.

[8] H. Zeng, H. Ma, L. Xu, J. Gao, M. Yan, Q. Wang, Int. J. Biol. Macromol. **2025**, 297, 139847.

[9] X. Li, Z. Wang, W. Bi, F. Yang, X. Wang, Y. Wang, H. Zhao, Poly **2024**, 309, 127465.

[10] M. Gong, X. Wang, Z. Wu, L. Yue, Q. Chen, H. Li, X. Lin, L. Zhang, D. Wang, Small. **2024**, 20, 2400161.

[11] Z. Li, F. Yin, W. He, T. Hang, Z. Li, J. Zheng, X. Li, S. Jiang, Y. Chen, Int. J. Biol. Macromol. **2023**, 230, 123117.

[12] Z. Sun, C. Dong, B. Chen, W. Li, H. Hu, J. Zhou, C. Li, Z. Huang, Small. **2023**, 19, 2303612.

[13] Y. Dong, N. Pan, M. Zhu, M. Tang, Y. Wu, Z. You, X. Zhou, M. Chen, Industrial Crops and Products **2022**, 187, 115491.

[14] Y. Gao, Y. Wang, Y. Dai, Q. Wang, P. Xiang, Y. Li, G. Gao, Eur. Polym. J. **2022**, 164, 110981.

[15] X. Duan, Y. Wang, T. Lei, Q. Zhang, A. Wan, Z. Xia, J. Fan, Int. J. Biol. Macromol. **2025**, 295, 139713.

[16] M. He, L. Chen, L. Zhang, L. Shen, H. Zhen, L. Wang, P. Xu, J. Bao, JMatS **2022**, 57, 16830-16841.

[17] S. Pan, C. Xiang, Z. Liu, G. Tong, C. Zhang, X. Zhu, Journal of Materials Chemistry C **2024**, 12, 8813-8824.

[18] X. Qin, Z. Zhao, J. Deng, Y. Zhao, S. Liang, Y. Yi, J. Li, Y. Wei, Carbohydr. Polym. **2024**, 335, 121920.

[19] Y. Hu, X. Maimaitiyiming, Int. J. Biol. Macromol. **2024**, 278, 134712.

[20] X. Li, L. Cao, L. Chen, Biochem. Eng. J. **2022**, 187, 108606.

[21] T. Lei, X. Duan, H. Zhao, S. Ma, X. Ma, N. Wang, Q. Zhang, A. Wan, Z. Xia, W. Shou, J. Fan, Chem. Eng. J. **2025**, 504, 158700.
